# Apoptotic Bodies Restore NAD and Mitochondrial Homeostasis in Fibroblasts

**DOI:** 10.1002/advs.202415691

**Published:** 2025-05-19

**Authors:** Shutong Qian, Siya Dai, Chunyi Guo, Wenjun Wang, Jiajia Pang, Yichen Shen, Mingyuan Xu, Jie Hu, Wenguo Cui, Xiaoming Sun, Jinghong Xu

**Affiliations:** ^1^ Department of Plastic Surgery The First Affiliated Hospital Zhejiang University School of Medicine 79 Qingchun Road Hangzhou 310003 P. R. China; ^2^ Department of Plastic and Reconstructive Surgery Shanghai Ninth People's Hospital Shanghai JiaoTong University School of Medicine 639 Zhi Zao Ju Road Shanghai 200011 P. R. China; ^3^ Department of Orthopaedics Shanghai Key Laboratory for Prevention and Treatment of Bone and Joint Diseases Shanghai Institute of Traumatology and Orthopaedics Ruijin Hospital Shanghai Jiao Tong University School of Medicine 197 Ruijin 2nd Road Shanghai 200025 P. R. China

**Keywords:** apoptotic body, mitophagy, NAD homeostasis, skin fibrosis

## Abstract

Fibrotic skin diseases are characterized by excessive fibroblast proliferation and pathological extracellular matrix deposition. As a pivotal coenzyme in cellular energetics, NAD homeostasis perturbation is implicated in fibrosis. Multiple studies have demonstrated the therapeutic potential of mesenchymal stem cells (MSCs) against cutaneous fibrosis, while the specific mechanism remains elusive. Herein, this work finds that although almost all MSCs undergo in situ apoptosis within 24 h post‐subcutaneous administration, MSC‐derived apoptotic bodies (ABs) mediated potent anti‐fibrotic effects. Mechanistically, ABs can restore NAD and mitochondrial homeostasis through NAMPT transfer, FOXO1 deacetylation enhancement, and PINK1/PARKIN‐dependent mitophagy activation. To achieve penetration into the hard matrix of fibrotic skin, permeable apoptotic bodies (pABs) are constructed via metabolic glycoengineering and copper‐free click chemistry techniques. In both keloid xenograft and scleroderma murine models, pABs can significantly penetrate collagen matrix and reduce skin fibrosis. In summary, this research establishes a highly promising strategy for reversing skin fibrosis with hard fibrotic matrix.

## Graphical abstract

Mesenchymal stem cell‐derived apoptotic bodies (MSC‐ABs) target keloid fibroblasts (KFs), restoring nicotinamide adenine dinucleotide (NAD) metabolism and mitochondrial function, suppressing collagen overproduction, and rebalancing tissue homeostasis, offering a novel therapy for keloid.

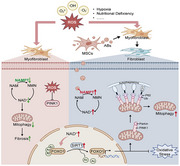

## 1 Introduction

Skin fibrosis is a pathophysiologic process characterized by dysregulated fibroblast proliferation and aberrant extracellular matrix (ECM) deposition.^[^ ^1^ ^]^ The most typical diseases are keloid and scleroderma, which affect millions of people worldwide. A hallmark of fibrotic progression is the transdifferentiation of fibroblasts into α‐smooth muscle actin (α‐SMA)⁺ myofibroblasts, and reactive oxygen species (ROS) is directly related to this process. Notably, keloid fibroblasts (KFs) exhibit significantly enhanced ROS production and antioxidant activity compared to normal fibroblasts (NFs).^[^ ^2^ ^]^ ROS overaccumulation can induce mitochondrial dysfunction and disrupt cellular redox homeostasis. Nicotinamide adenine dinucleotide (NAD/NAD⁺), the central redox cofactor for oxidoreductases, plays an irreplaceable role in regulating cellular energy metabolism. Multiple studies have shown that the deficiency of NAD^+^ is closely related to fibrosis, and the supplementation of NAD^+^ or its precursor substances can alleviate mitochondrial dysfunction.^[^ ^3^, ^4^ ^]^ Thus NAD⁺‐centric interventions have begun to be applied to various diseases.^[^ ^5^, ^6^ ^]^ Fibrosis is a highly dynamic process, wherein targeting its metabolic plasticity offers therapeutic promise. Consequently, restoring NAD⁺ homeostasis to inhibit fibroblast activation represents a critical challenge in combating skin fibrosis.

Mesenchymal stem cells (MSCs) have demonstrated capacity to modulate redox equilibrium and mitochondrial function in myofibroblasts.^[^ ^7^, ^8^, ^9^ ^]^ Paradoxically, many studies have found that >90% of transplanted MSCs undergo rapid in situ apoptosis within 24 h post‐administration, yet retain therapeutic efficacy through apoptotic derivatives.^[^ ^10^, ^11^ ^]^ Mesenchymal stem cell‐derived apoptotic bodies (MSC‐ABs), as apoptotic products of MSCs, have been reported to participate in various pathophysiologic processes including wound healing,^[^ ^12^, ^13^, ^14^, ^15^ ^]^ osteogenesis,^[^ ^16^ ^]^ and post‐infarct cardiac remodeling.^[^ ^17^ ^]^ However, the exact effect and molecular mechanism of MSCs‐ABs on skin fibrosis remain poorly characterized. Herein, we find that MSCs‐ABs can function as therapeutic mediators for skin fibrosis by improving NAD homeostasis by delivering nicotinamide phosphoribosyltransferase (NAMPT), promoting mitophagy in fibroblasts, and reversing the fibrotic phenotype.

**Scheme 1:**
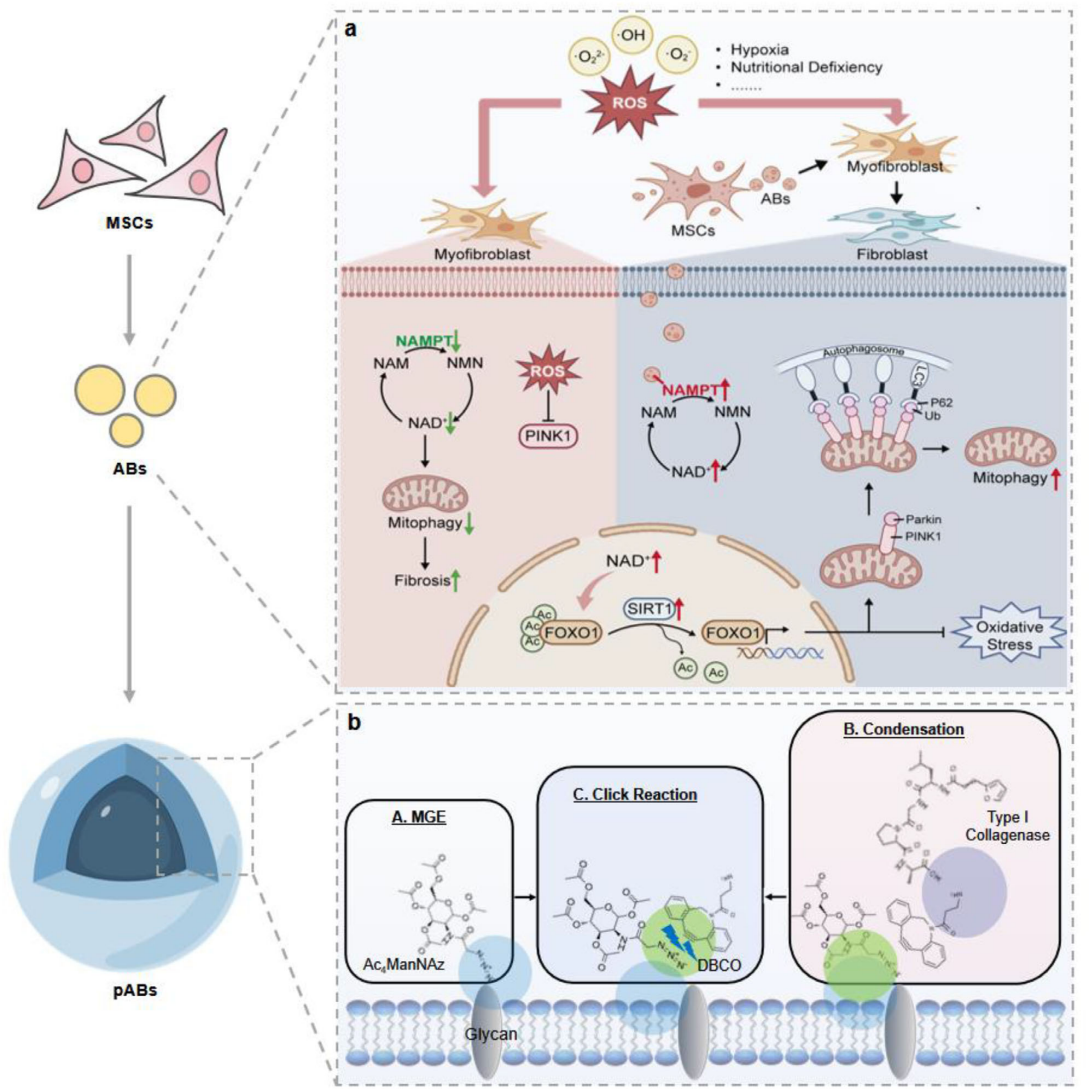
a) Molecular mechanism diagram of ABs promoting mitochondrial autophagy in KFs. b) Fabrication process of permeable ABs (pABs).

Notably, fibrotic skin fibroblasts are typically encased in ECM collagen from activated myofibroblasts, creating structural barriers that impede nanotherapeutic penetration and hinder NAD⁺ homeostasis modulation.^[^ ^18^ ^]^ Type‐I collagenase can specifically hydrolyze the 3D helical structure of extracellular matrix collagen, thus improving uptake efficiency.^[^ ^19^, ^20^, ^21^, ^22^ ^]^ Herein, we constructed permeable apoptotic bodies (pABs) via metabolic glycoengineering and copper‐free click chemistry techniques. Azide‐modified mannose (Ac_4_ManNAz) was first used to endow the membrane surface of MSCs with azide groups (‐N_3_). Then, DBCO‐NH_2_ was polymerized through amide reaction to bind type I collagenase. Finally, pABs were obtained by modifying type‐I collagenase onto the cell membrane surface using copper‐free click chemistry technology. Engineered pABs can penetrate layers of collagen fibers, search for activated myofibroblasts, improve NAD homeostasis in fibroblasts within 1 week, and finally reverse the fibrotic phenotype (**Scheme** **1**).

## 2 Results

### 2.1 ADSCs Rescue Redox‐NAD⁺ Axis Dysregulation in Fibrotic Skin

**Figure 1:**
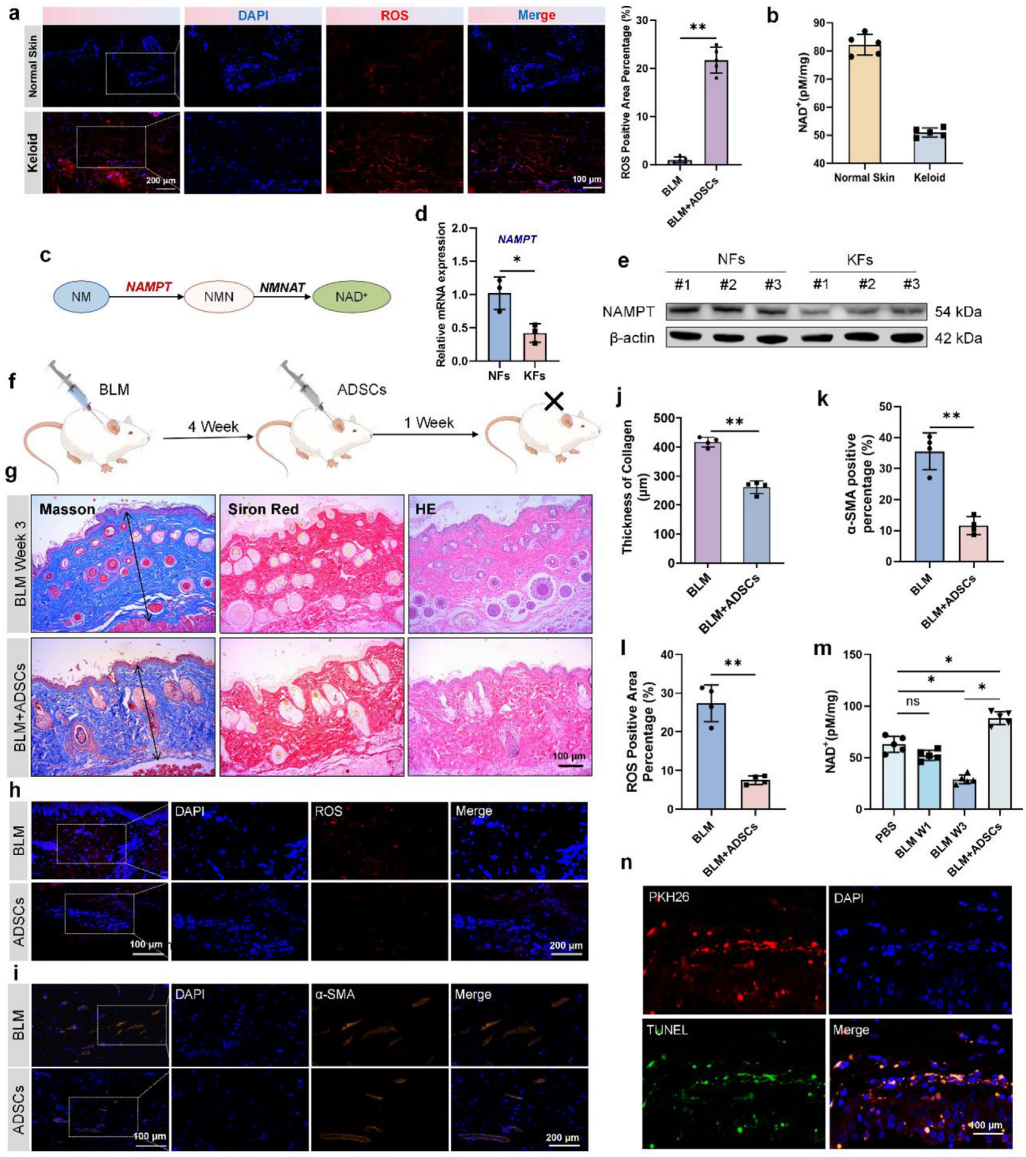
ROS accumulation and NAD metabolism disorder in fibrotic skin. a) Typical ROS staining images and corresponding positive area statistical analysis of scar tissue and normal skin tissue (n = 5). Scale bars, 100 µm. b) Comparison of NAD^+^ levels between keloid and normal skin (n = 5). c) Schematic of NAD synthesis. d–e) Comparison of the mRNA (n = 3) and protein content of NAMPT in NFs and KFs. f) Animal experiment schematic diagram. g) Typical images of HE, Masson, and Siron Red staining. Scale bars, 100 µm. h,i) Typical images of ROS staining and α‐SMA IF staining. Scale bar, 200 µm. j–l) Comparison of collagen thickness, ROS positive area, and α‐SMA positive area (n = 4). m) NAD^+^ levels of different group (n = 5). n) Typical images of PKH26 labeled ADSCs co‐stained with in situ TUNEL. Scale bars, 100 µm. * *p* < 0.05, **p < 0.01.

Oxidative stress‐mediated ROS accumulation and NAD⁺ metabolic dysregulation both contribute to skin fibrosis.^[^ ^23^, ^24^, ^25^ ^]^ To evaluate the level of oxidative stress and NAD^+^, ROS staining and NAD^+^ level tests were performed. The results showed that compared to normal skin, the expression of ROS in keloid was significantly increased (**Figure** **1**a) while NAD^+^ level significantly decreased (Figure 1b). The expression levels of NAMPT – the key enzyme protein involved in NAD metabolism – in KFs significantly decreased compared to NFs (Figure 1c,d,e). Multivariate Spearman analysis confirmed strong inverse correlation between NAD⁺ bioavailability and ROS accumulation in both human keloid (r = −0.9407, *p* = 0.0172) and bleomycin‐induced murine fibrotic models (r = −0.9632, *p* = 0.0368) (Figure S1a,b, Supporting Information). These findings collectively demonstrate impaired NAD⁺ metabolism accompanied by redox imbalance in fibrotic tissues.

Multiple basic research and clinical trials have established MSCs as promising antifibrotic agents.^[^ ^26^, ^27^ ^]^ In this research, bleomycin‐induced fibrotic murine models received bilateral subcutaneous injections of 1 × 10⁶ adipose‐derived stem cells (ADSCs) at lesion sites (Figure 1f). HE staining showed that compared with the group treated with bleomycin and phosphate buffered saline, collagen thickness and deposition were significantly down‐regulated in ADSCs group. Masson staining also showed a decrease in collagen deposition and densification, indicating a significant improvement in skin fibrosis in the ADSCs group (Figure 1g,j). ROS staining showed that compared with the control group, the ROS accumulation phenomenon was significantly improved in the ADSCs group, so as the NAD^+^ level. (Figure 1h,l,m). Immunofluorescence staining of α‐SMA also showed a significant decrease in fibrosis degree in the ADSCs group (Figure 1i,k). The above results indicated that the implantation of ADSCs had a great therapeutic effect on skin fibrosis.

Once MSCs are implanted, whether through local injection or systemic delivery, partial or even complete apoptosis often occurs within 24 h. In this study, through PKH26 fluorophore labeling coupled with TUNEL co‐staining, we observed that almost all ADSCs undergo in situ apoptosis after 24 h of implantation (Figure 1n). This may be due to the cytotoxic effects of many inflammatory cells in the local microenvironment of fibrotic skin on ADSCs. However, as mentioned above, 1 week after the implantation of ADSCs, tissue staining still demonstrated the therapeutic effect on skin fibrosis. These findings established MSC apoptosis as an essential mechanistic prerequisite for therapeutic efficacy in skin fibrosis.

### 2.2 MSCs‐ABs Improve Mitochondrial and NAD Homeostasis of KFs

**Figure 2:**
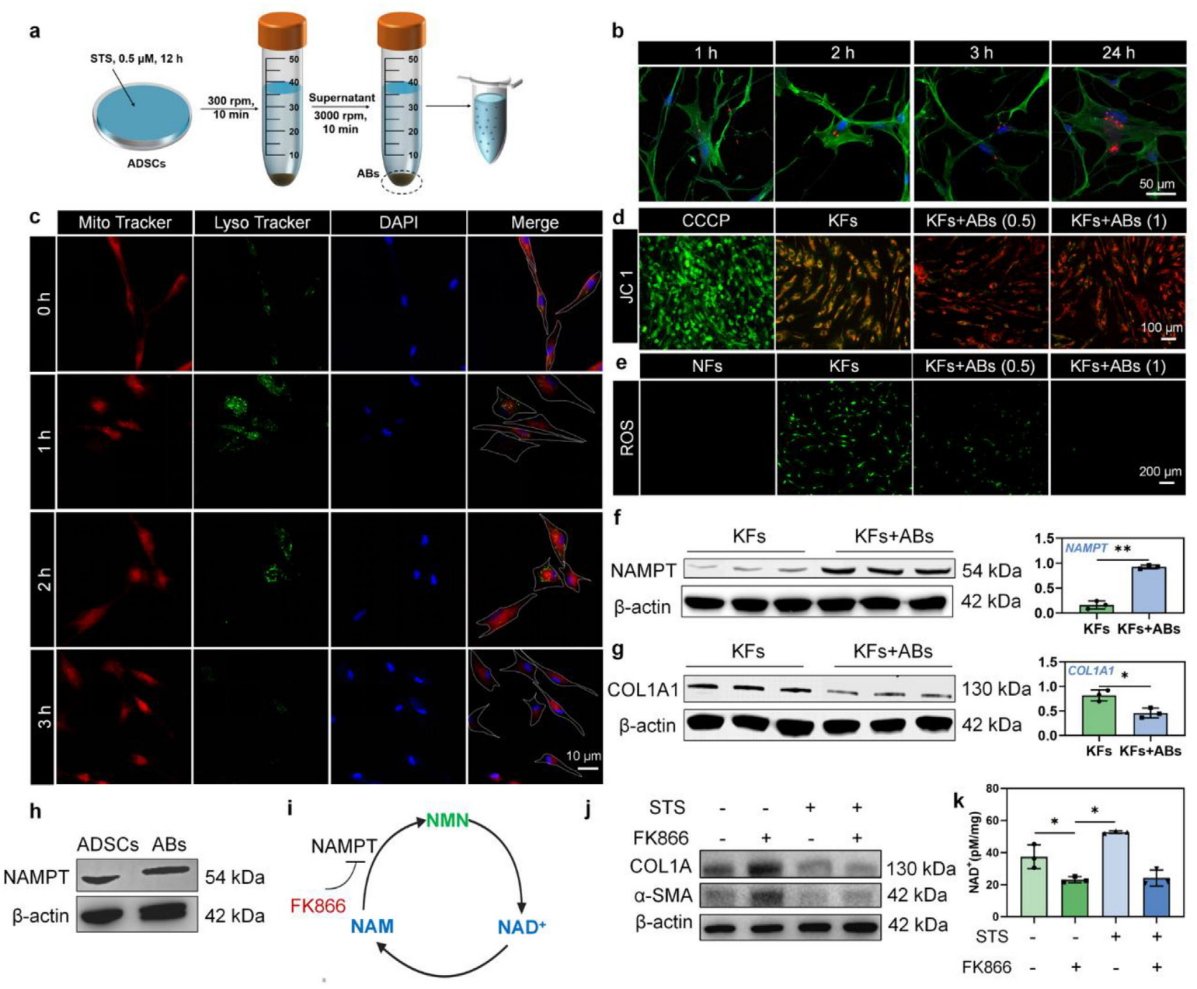
ABs promote NAD metabolism and mitophagy in fibroblasts and reverse fibrotic phenotype. a) Preparation schematic of ABs. b) Typical images of KFs uptaking ABs at different time points. Scale bars, 50 µm. c) Mitophagy process of KFs co‐cultured with ABs at different time points. Scale bars, 10 µm. d) Typical images of JC‐1 staining of KFs co‐cultured with ABs. Scale bars, 100 µm. e) Typical images of ROS staining of KFs co‐cultured with ABs. Scale bars, 200 µm. f,g) Typical Western Blot images and protein expression analysis of NAMPT (n = 3) and COL1A1 (n = 3) in KFs co‐cultured with KFs. h) Typical Western Blot images of NAMPT in ADSCs and ABs. i) Schematic of NAD metabolism. j) FK866 reversed the anti‐fibrotic phenotype of KFs. k) Comparison of NAD^+^ levels in KFs co‐cultured with ABs (n = 3). *p < 0.05, **p < 0.01.

To investigate the influence and mechanism of MSCs‐ABs on fibroblast fibrosis phenotype modulation, we generated apoptotic bodies via staurosporine‐induced apoptosis (**Figure** **2**a). Transmission electron microscope (TEM) showed the double‐layer cell membrane of ABs, and the structure was intact (Figure S2a, Supporting Information). Dynamic light scattering (DLS) test revealed that the diameter of ABs is about 600–1000 nm, and the zeta potential is −15 mV (Figure S2b,c, Supporting Information). Cell‐Counting‐Kit‐8 (CCK‐8) test showed that when co‐cultured with ABs of 1 µg mL^−1^, the cellular biocompatibility was highest, while as the concentration increased, the cell survival rate decreased (Figure S2d, Supporting Information). Therefore, in subsequent cell experiments, we selected the concentration of 1 µg mL^−1^. Western blot confirmed that MSC‐ABs retained parental ADSC marker CD105, and apoptosis marker proteins caspase‐3 and cleaved caspase‐3 (Figure S2e, Supporting Information). The cell uptake results (Figure 2b) showed that as time increased, the amount of ABs uptake by KFs increased. At the same time, the cell morphology began to shrink and gradually became slender. In addition, in vitro wound healing and trans‐well migration and invasion experiments showed that the treatment of ABs significantly reduced the cell migration and invasion abilities of KFs (Figure S3a–e, Supporting Information).

Mitophagy is a process in which damaged mitochondria are specifically encapsulated in autophagosomes and fused with lysosomes to complete lysosomal degradation under ROS stress stimulation, providing essential quality control for mitochondrial homeostasis and cellular redox balance. Defective mitophagic clearance is an important cause of fibrosis.^[^ ^28^, ^29^ ^]^ Herein, we investigated the effects of MSCs‐ABs on mitochondrial function and mitophagy of KFs. First, co‐staining of lysosomes and mitochondria was performed to detect the effect of ABs on mitophagy. With the addition of MSCs‐ABs, the co‐localization of mitochondria and lysosomes gradually increased (Figure 2c), peaking at 2 h post‐treatment before resolving by 3 h. The cell morphology started with a long and spindle‐like shape, gradually expanded, and then shrank and curled into a slender shape. The above results indicated that MSCs‐ABs can significantly promote mitophagy of KFs. In addition, JC‐1 staining showed that MSCs‐ABs contributed to a concentration‐dependent increase in cell mitochondrial membrane potential (Figure 2d). Besides, ROS level in KFs was significantly reduced with the addition of MSCs‐ABs (Figure 2e). These coordinated responses establish MSC‐ABs as potent inducers of mitochondrial homeostasis.

Given mammalian NAD⁺ biosynthesis occurs through three evolutionarily conserved routes^[^ ^30^ ^]^ (Figure S4a, Supporting Information), we first verified the dominant NAD⁺ biosynthetic route in KFs using pathway‐specific precursors: β‐nicotinamide mononucleotide (salvage), quinolinic acid (de novo), and nicotinic acid (Preiss‐Handler). The results showed that nicotinamide mononucleotide significantly up‐regulated NAD^+^ level in KFs, while quinolinic acid and nicotinic acid had no significant effect. To further validate the effect of the salvage pathway on NAD metabolism in KFs, we treated KFs with NAMPT inhibitor FK866 (Figure 2i). A significant decrease was shown in NAD^+^ level (Figure S4b,c, Supporting Information). The above results demonstrated that the salvage synthesis pathway is the key pathway for NAD metabolism in KFs. In addition, we added nicotinamide mononucleotide and NAMPT recombinant protein to KFs, western blotting result showed a decrease in the expression of α‐SMA protein in KFs, indicating that enhanced NAD metabolism leads to the anti‐fibrotic effect (Figure S4d, Supporting Information).

There was a significant increase in protein expression of NAMPT and a decrease in collagen I (COL1A1) in KFs after treatment with MSCs‐ABs (Figure 2f,g). MSCs‐ABs can inherit the NAMPT protein from MSCs (Figure 2h). To verify whether MSCs‐ABs inhibit fibrosis by up‐regulating NAMPT, we first added FK866 to ADSCs and induced apoptosis through staurosporine to obtain MSCs‐ABs. After co‐culturing with KFs, the protein expression levels of α‐SMA and COL1A1 were detected. The fibrotic inhibitory effect of MSCs‐ABs can be reversed by FK866 (Figure 2j). In addition, the NAD^+^ level in KFs treated with ABs were increased, which can also be reversed by FK866 (Figure 2k). The above results indicated that ABs can up‐regulate the NAD salvage synthesis pathway of KFs by increasing expression of NAMPT, thereby promoting NAD metabolism.

### 2.3 MSCs‐ABs Promote Mitophagy in KFs through NAMPT/SIRT1/FOXO1 Pathway

**Figure 3:**
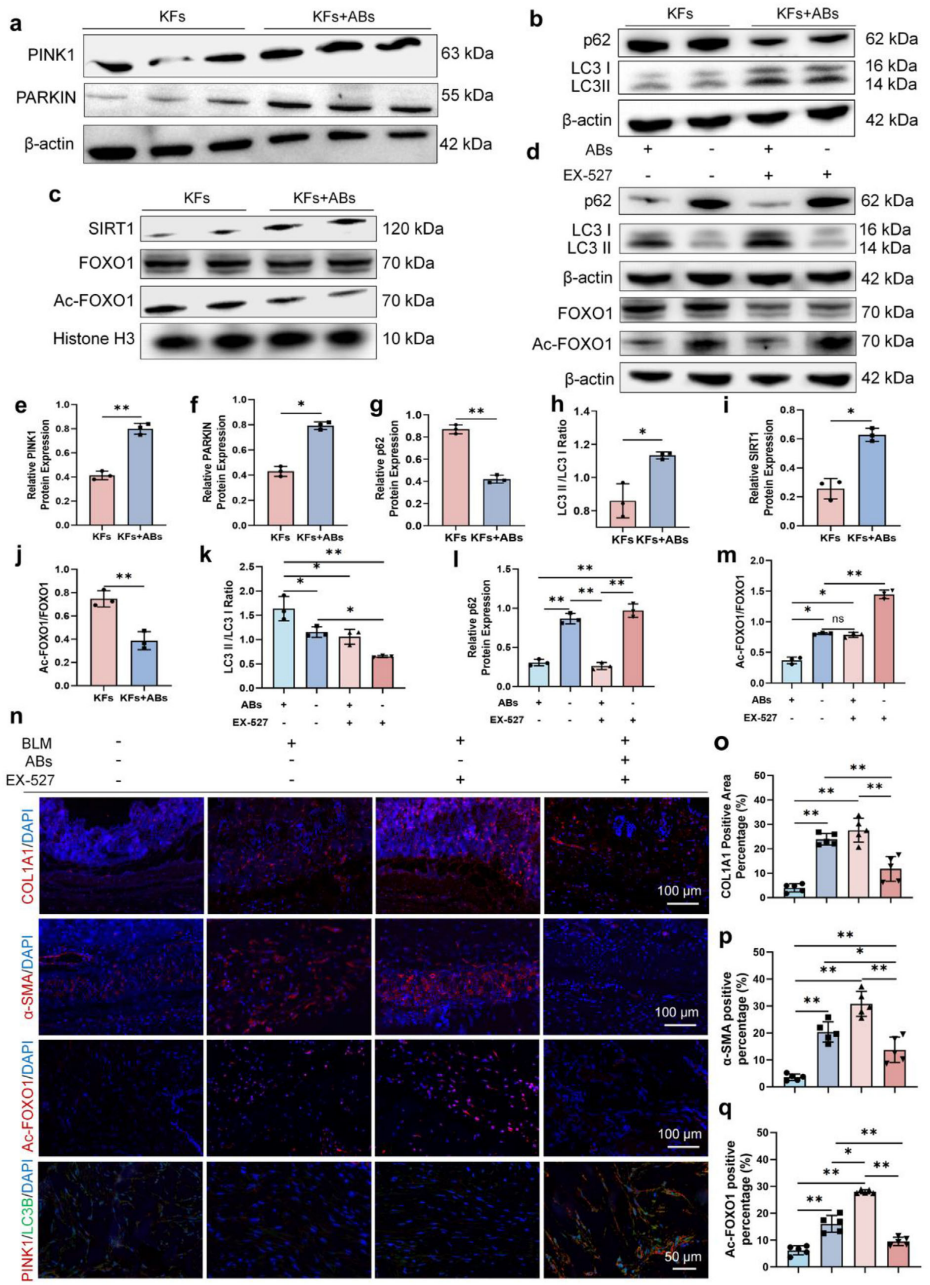
MSCs‐ABs promote mitophagy in KFs through NAMPT/SIRT1/FOXO1 pathway. a–c) Typical western blot image of PINK1, PARKIN, p62, LC3 I/II, SIRT1, FOXO1, Ac‐FOXO1 in KFs and KFs treated with ABs. e–m) Statistical analysis of PINK1, PARKIN, p62, LC3 I/II, SIRT1, FOXO1, Ac‐FOXO1 in KFs and KFs treated with ABs (n = 3). j) Typical western blot image of p62, LC3 I/II, FOXO1, Ac‐FOXO1 in different groups. k‐m) Statistical analysis of p62, LC3 I/II, FOXO1, Ac‐FOXO1 in different groups (n = 3). n–p) Immunofluorescence staining and statistical analysis of α‐SMA, COL1A1, Ac‐FOXO1 and LC3B/PARKIN in different groups (n = 5). *p < 0.05. **p < 0.01.

The PINK1/PARKIN axis is the main pathway for identifying damaged mitochondria in the process of mitophagy. After the treatment with MSCs‐ABs, the protein expression of PINK1 and PARKIN in KFs significantly increased (**Figure** **3**a,e,f). To further determine whether the decrease in PINK1 protein expression in mitochondria is due to lysosomal improvement of mitophagy fragment accumulation or inhibition of the PINK1/PARKIN pathway, we investigated the LC3 II/LC3 I ratio and p62 in KFs. During autophagy initiation, LC3 degrades from protein complex LC3 I to LC3 II, which is then recruited to autophagosomes and interacts with p62. The results showed that after treatment with MSCs‐ABs, the protein expression of LC3 II/LC3 I significantly increased, while p62 significantly decreased (Figure 3b,g,h). The above results indicated that MSCs‐ABs can induce an increase in mitophagy by up‐regulating the PINK1/PARKIN pathway rather than impairing lysosomal degradation capacity, thereby restoring mitochondrial quality control.

NAD metabolism is involved in the deacetylase activity of various NAD‐dependent proteins in mitochondria, particularly the sirtuins family.^[^ ^31^, ^32^ ^]^ Among these, SIRT1 emerged as the predominant regulator of mitochondrial homeostasis, with MSCs‐ABs significantly increasing SIRT1 but decreasing SIRT3 expression (Figure 3c). However, the expression of SIRT3 significantly decreased (Figure S5a, Supporting Information). and SOD2, a downstream gene of SIRT3 showed no difference (Figure S5b, Supporting Information). Therefore, it can be considered that SIRT1 is a key gene for MSCs‐ABs to promote mitochondrial homeostasis. Mechanistically, NAD⁺ enhances mitochondrial function through SIRT1‐mediated FOXO1 deacetylation, which activates PINK1/PARKIN‐dependent mitophagy.^[^ ^33^, ^34^, ^35^ ^]^ To explore the link between mitophagy and the SIRT1‐FOXO1 axis after treatment of MSCs‐ABs, we evaluated the protein expression of SIRT1 in KFs, as well as the deacetylation level of FOXO1. Compared with the control group, the SIRT1 protein level in KFs treated with ABs was significantly enhanced, while the Ac‐FOXO1 protein level was significantly reduced (Figure 3c,i,j). Pharmacological SIRT1 inhibition (EX527) abolished FOXO1 deacetylation (Figure 3d) and attenuated MSC‐AB‐induced mitophagic flux (Figure 3k–m). In bleomycin‐induced fibrotic murine models, MSC‐AB administration reversed SIRT1 inhibition‐aggravated fibrosis, promoting mitophagy and normalizing α‐SMA, COL1A1 and Ac‐FOXO1 expression. These results indicated that MSCs‐ABs can enhance mitophagy and treat skin fibrosis mediated by the PINK1/PARKIN pathway through the NAMPT/SIRT1/FOXO1 axis.

### 2.4 Regulatory Effect of MSCs‐ABs on Gene Transcription in KFs

**Figure 4:**
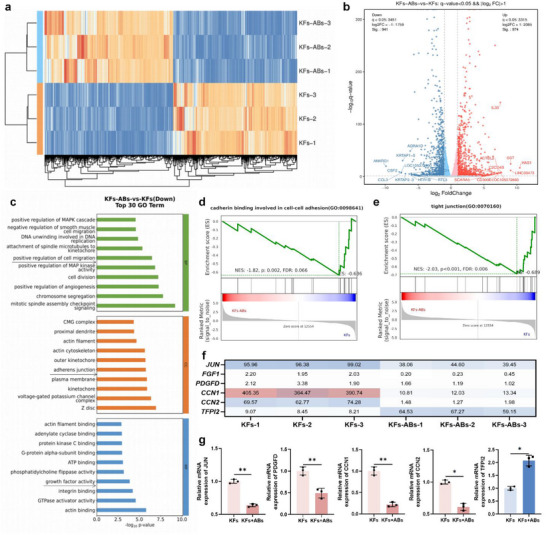
ABs regulate the expression of fibrotic related genes. a,b) Heatmap and Volcano maps. c,d) GSEA diagram of cross‐linking of cell adhesion and tight junction. e) Top 30 GO terms of down‐regulated genes. f,g) Cluster heatmap and qRT‐PCR validation of genes related to pro‐fibrotic or anti‐fibrosis effect (n = 3). *p < 0.05, **p < 0.01.

Ribonucleic acid (RNA) sequencing of AB‐treated KFs revealed extensive transcriptomic remodeling. Sample quality assessment showed a high degree of dispersion between groups and a high correlation coefficient within each group (Figure S6a–d, Supporting Information). There were 974 up‐regulated differential expression gene (DEGs) and 941 down‐regulated DEGs in KFs treated with ABs (**Figure** **4**a,b). Gene Ontology (GO) enrichment analysis of downregulated DEGs highlighted suppression of profibrotic pathways: cell migration, focal adhesion, and growth factor binding (Figure 4c). Gene Set Enrichment Analysis (GSEA) confirmed negative regulation of cell adhesion and tight junction assembly (Figure 4d,e). In addition, pro‐fibrotic genes such as cellular communication network factor 1 (CCN1), CCN2, Jun proto‐oncogene (JUN), platelet‐derived growth factor D (PDGFD) and fibroblast growth factor 1 (FGF1) were significantly decreased, while anti‐protic gene such as tissue factor pathway inhibitor 2 (TFPI2) was significantly increased. The above results were also validated by quantitative real‐time PCR (qPCR) (Figure 4f,g). The above results further indicated that ABs possess therapeutic effect against skin fibrosis by regulating cell migration and adhesion connections.

### 2.5 Fabrication and Characterization of pABs

**Figure 5:**
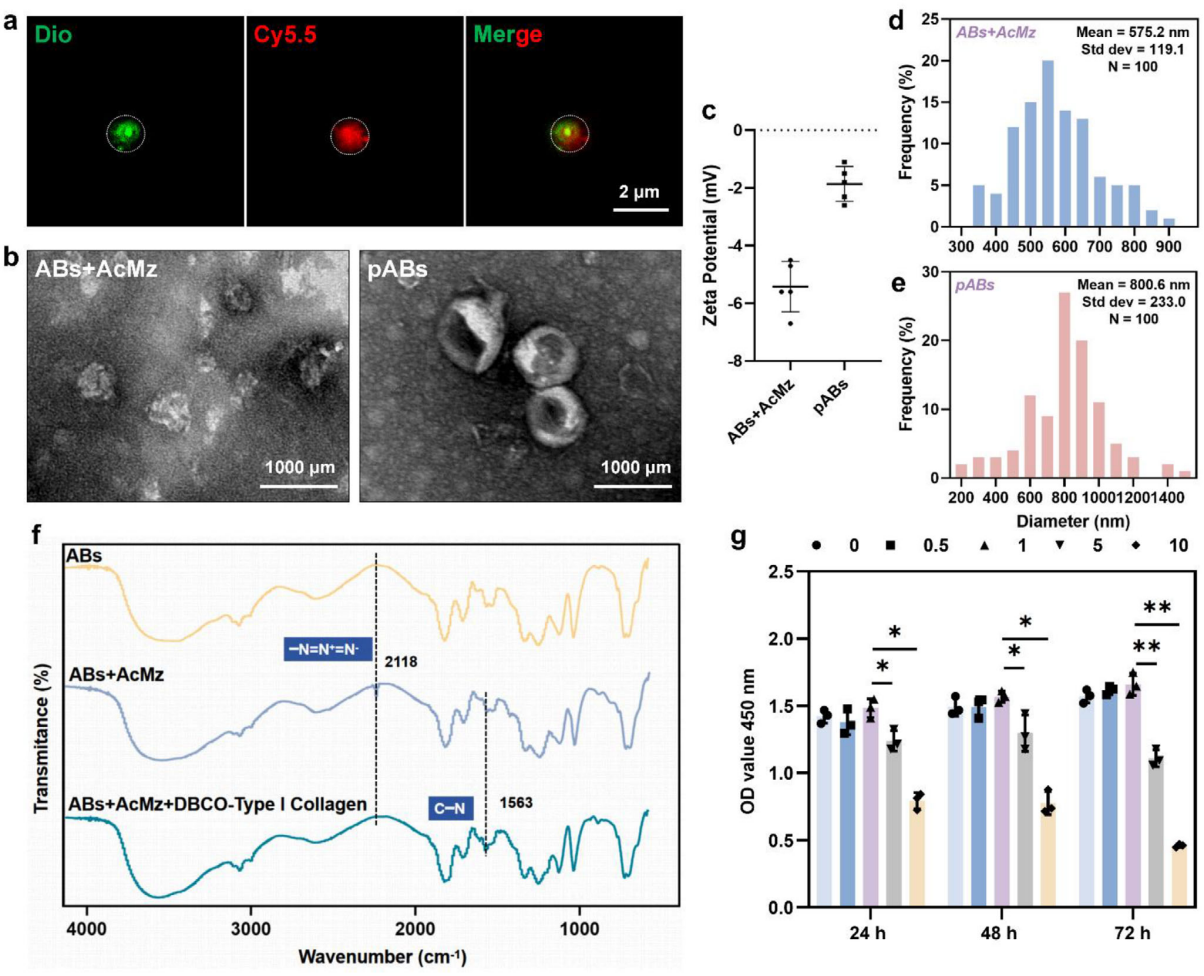
Preparation and characterization of pABs. a) Typical images of DBCO (Dio) binding to AcMz (Cy5.5) on cell membrane surface. Scale bars, 2 µm. b) Typical TEM images of AcMz‐treated ABs and pABs. Scale bars, 10 µm. c–e) Comparison of Zeta potentials and particle size distribution of AcMz treated ABs and pABs (n = 5). f) Infrared spectral images of ABs, ABs+AcMz, and pABs. g) Cytotoxicity testing of co cultured fibroblasts with different concentrations of pABs (n = 3). * *p* < 0.05, **p < 0.01.

The dense collagen architecture of fibrotic skin creates formidable biomechanical barriers to therapeutic penetration. To address this, we engineered permeable apoptotic bodies (pABs) via metabolic glycoengineering and copper‐free click chemistry (Figure S7, Supporting Information). First, DBCO was labeled with anthocyanins, and Ac4ManNAz modified ABs (termed as AcMz‐ABs) was labeled with DiR. The results showed that DBCO overlapped with AcMaz‐ABs (**Figure** **5**a). TEM showed that after adding AcMz, the surface of ABs was covered with Ac4ManNAz and showed an appearance of wrinkled vesicles. After binding with DBCO‐type 1 collagenase, the surface of pABs became smooth and its diameter increased (Figure 5b). Compared to AcMaz‐ABs, the zeta potential of pABs increased from −5.8 to −2.0 mV (Figure 5c), and the diameter significantly increased, increasing from 575.2 ± 119.1 nm to 800.5 ± 233.0 nm (Figure 5d,e). Fourier transform infrared spectrometer (FTIR) showed that after co‐incubation of AzMz‐ABs, the characteristic peak of azide groups (2118.8 cm^−1^) appeared, and disappeared after adding DBCO, proving the successful click chemical reaction between AcMz and DBCO. In addition, after the amide condensation reaction between DBCO and type I collagenase, the characteristic peak of primary amide bonds (1565 cm^−1^) was detected in pABs. This further demonstrated the successful combination of DBCO‐type 1 collagenase and Ac4ManNAz (Figure 5f). We further tested the biocompatibility of pABs, the results showed that when the concentrations were 0.5 and 1 µg mL^−1^, the cell survival rate was greater than 100%, supporting clinical translatability (Figure 5g).

### 2.6 pABs can Penetrate Deep into the Matrix In Vivo

**Figure 6:**
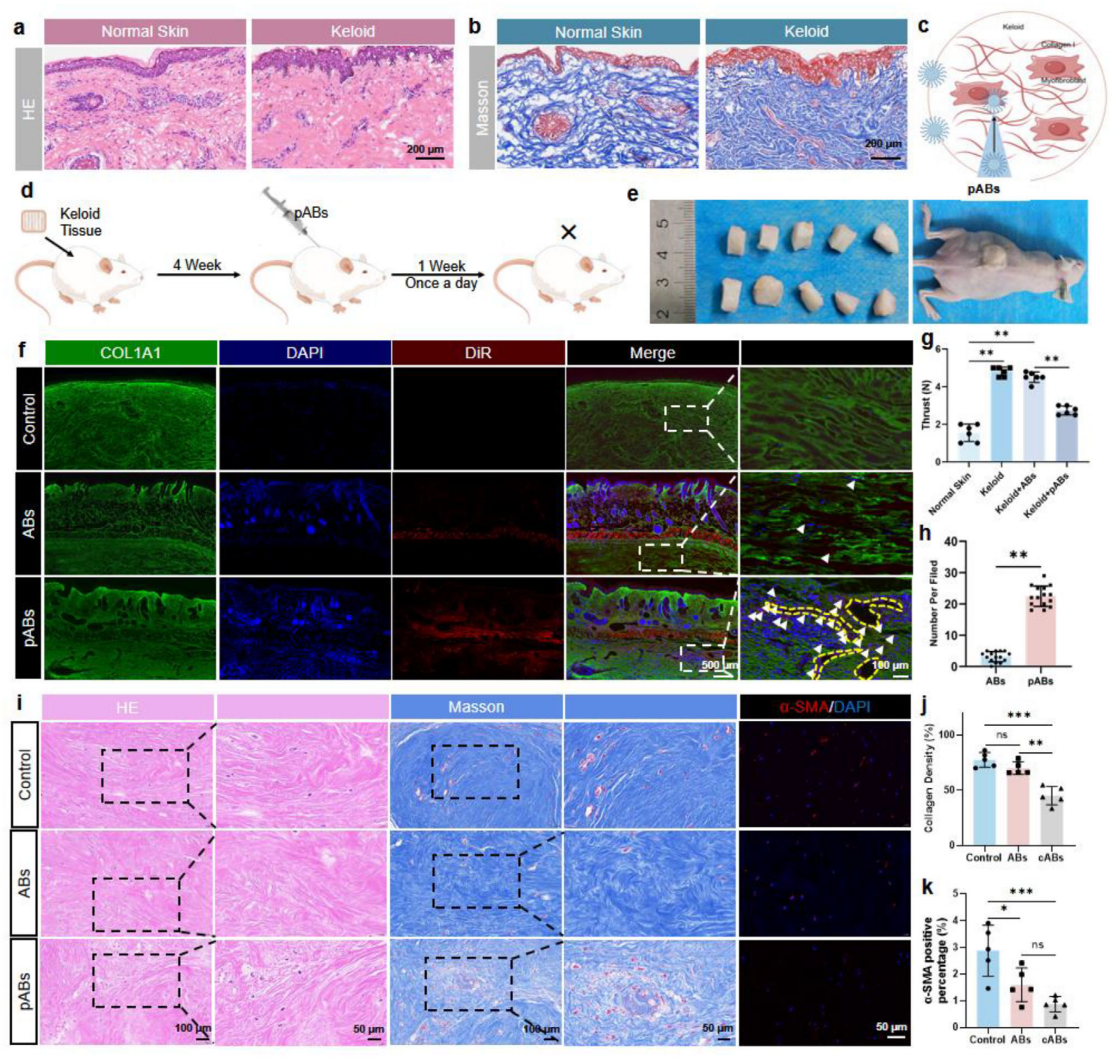
pABs promote the infiltration and delivery to fibrotic matrix. a,b) Typical images of HE and Masson staining of normal skin and keloid. Scale bars, 200 µm. c) A brief scheme of pABs in treating skin fibrosis. d) A brief scheme of a nude mouse keloid transplantation model. e) Keloid nude mouse transplantation model. f) Typical images of DiR labeled ABs and pABs in the process of scar tissue infiltration. Scale bars, 500 µm (left), 100 µm (right). g) Comparison of injection thrust between normal tissue, keloid and keloid treated with ABs and pABs (n = 6). h) Comparison of the number of ABs and pABs infiltrating keloid (n = 15). i) Typical HE and Masson staining images of different groups. j,k) Comparison of collagen density and α‐SMA positive percentage between different groups. *p < 0.05, **p < 0.01, ***p < 0.001.

Compared with normal skin tissue, fibrotic tissue often has dense and deformed collagen fibers, which hinder the penetration of therapeutic substances into the diseased tissue (**Figure** **6**a,b). Engineered pABs can enzymatically remodel fibrotic ECM through surface‐anchored collagenase activity, enabling deep tissue penetration. To verify the therapeutic effect of pABs on skin fibrosis, DiR‐labeled pABs were injected into the back of human keloid xenograft models (Figure 6c,e). pABs can penetrate the collagen layer and form a sinus through the matrix, achieving the delivery of ABs deep into the keloid matrix (Figure 6f). Besides, compared to normal skin, the keloid group required 2.5 times the thrust to inject to the same level. After adding pABs, the required thrust significantly decreased by half of the ABs group (Figure 6g). The number of ABs/pABs in each group was counted, and the results showed that the permeability of pABs was much better than that of ABs (Figure 6h). Therefore, it can be concluded that the permeability of pABs was greatly enhanced compared with ABs, allowing them to penetrate deep into the matrix in vivo. HE, Masson and α‐SMA immunofluorescence staining showed the great therapeutic effect of ABs and pABs on keloid transplantation model (Figure 6i). Compared with the control group and the ABs group, the collagen density in pABs group was significantly decreased, indicating that pABs could penetrate to the matrix depth and promote collagen degradation (Figure 6j). The area analysis of α‐SMA positive percentage showed that ABs and pABs both had anti‐fibrosis effect (Figure 6k). Inflammatory factors and transforming growth factor‐β 1 (TGF‐β1) can also indicate improvements in skin fibrosis. Therefore, we used mouse transplantation model and bleomycin‐induced skin fibrosis model, and performed qPCR analysis on skin tissues to assess the expression levels of interleukin‐6 (IL‐6), tumor necrosis factor‐α (TNF‐α), and TGF‐β1. The results showed that both ABs and pABs can down‐regulate the expression of these factors at the transcriptional level (Figure S8, Supporting Information). In conclusion, the above results demonstrated the excellent permeability and anti‐fibrosis effect of pABs.

### 2.7 pABs Improve NAD Metabolism and Treat Skin Fibrosis In Vivo

**Figure 7:**
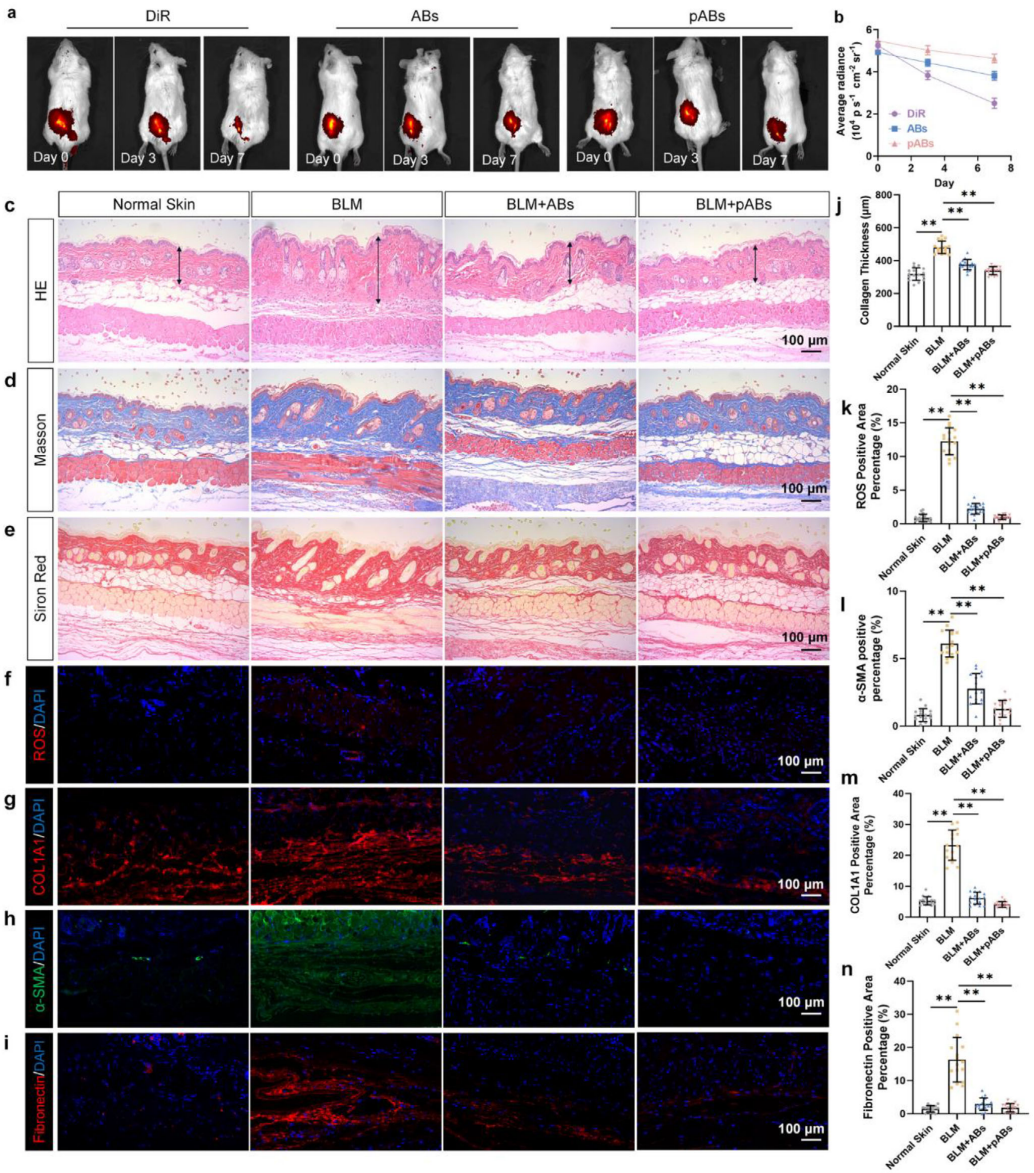
pABs significantly improved BLM induced skin fibrosis. a,b) Typical infrared imaging images and fluorescence signal intensity analysis of DiR labeled ABs or pABs injected into a mouse model of skin fibrosis. (n = 3) c–e) Typical images of HE, Masson, and Sirius red staining in each group. Scale bars, 100 µm. f) Typical images of in situ ROS staining in each group. Scale bars, 100 µm. g–i) Typical images of COL1A1, α‐SMA, and Fibronectin IF staining in each group. Scale bars, 100 µm. j) Thickness of collagen deposition in each group (n = 16). k) Percentage of ROS positive areas in each group (n = 16). l–n) Percentage of COL1A1, α‐SMA, and Fibronectin IF staining positive areas in each group (n = 16). *p < 0.05, **p < 0.01.

To verify the ability of pABs to treat skin fibrosis in vivo, we constructed bleomycin induced skin fibrosis mouse model and subcutaneously injected pABs daily for 1 week. Through infrared imaging of skin fibrosis mice treated with pABs, it was found that compared to the ABs and DiR groups, pABs exhibited prolonged tissue retention (**Figure** **7**a,b), thus exerting a more long‐lasting therapeutic effect.

The inhibitory effect on skin fibrosis of pABs was also investigated. HE and Masson staining showed that compared with the bleomycin group, both the ABs and pABs groups showed a significant decrease in collagen deposition and thickness (Figure 7c,d,j). Sirius red staining showed that compared with the bleomycin group, both the ABs and the pABs group exhibited anti‐fibrotic therapeutic effects. The expression of type I collagen, α‐SMA, and fibronectin increased, while pABs significantly decreased (Figure 7g–i,l–n). Tissue ROS staining reveals that compared with the bleomycin group, both the ABs and pABs groups significantly reduced ROS levels in bleomycin fibrosis mice (*p*<0.01) (Figure 7f,k). The above results demonstrated that pABs can enhance permeability deep into the fibrotic matrix, thereby promoting local NAD metabolism, reducing oxidative stress, and inhibiting fibrosis in vivo.

## 3 Discussion

As for the treatment of skin fibrosis, the current mainstream approaches predominantly target myofibroblast proliferation and apoptosis, yet fail to address the pathological plasticity of fibrotic phenotypes. Emerging evidence implicates fibroblast metabolic dysregulation as a critical driver of fibrogenesis, positioning metabolic reprogramming as a viable therapeutic strategy. NAD homeostasis disorder contributes to various fibrotic diseases.^[^ ^36^, ^37^ ^]^ In addition, mesenchymal stem cell therapies demonstrate anti‐fibrotic efficacy through NAD metabolic regulation.^[^ ^38^ ^]^ Our findings revealed MSC‐ABs can rectify NAD dyshomeostasis in fibrotic fibroblasts via targeted NAMPT delivery. It is worth noting that the role of NAMPT varies greatly in existing studies.^[^ ^39^, ^40^, ^41^ ^]^ This may be due to differences in the dosage and distribution of NAMPT. Intracellular NAMPT is a key enzyme in the NAD salvage synthesis pathway, while extracellular NAMPT is an inflammatory factor by driving macrophage polarization.^[^ ^42^ ^]^ In addition, excessive NAMPT can lead to leakage of NAMPT from cells, resulting in excessive accumulation of NAD metabolites, and thus producing the opposite effect of promoting inflammation and fibrosis.^[^ ^43^ ^]^ To circumvent these limitations, we engineered MSC‐ABs for precision NAMPT delivery. At the same time, we chose a concentration of 1 µg mL^−1^ of MSCs‐ABs for in vitro experiments, and 5 mg kg^−1^ for in vivo experiments. The safety and therapeutic effect of this concentration were verified through western blot and tissue staining. Therefore, for the first time, we confirmed the therapeutic effect of MSCs‐ABs on skin fibrosis by delivering NAMPT into cells and provided a new strategy for metabolic therapy of skin fibrosis.

NAD directly participates in regulating the activity and expression of Sirtuins protein family members, among which SIRT1 and SIRT3 are mainly involved in maintaining mitochondrial redox homeostasis. SIRT1 is the most prominent member, widely involved in cellular aging, metabolism, DNA damage repair, and immune regulation. SIRT3 is known as the main deacetylase in mitochondria, which can directly bind to SOD2 and inhibit cellular oxidative stress response. In this study, the treatment of MSC‐ABs induced significant upregulation of SIRT1 expression while downregulating SIRT3, with no observable changes in SOD2 protein levels. The observed inverse expression pattern between SIRT1 and SIRT3, potentially mediated by NAD regulation, suggests a possible negative regulatory relationship that warrants further investigation.

The therapeutic efficacy for fibrotic skin is significantly compromised by the excessively thick and dense collagen fiber matrix. In this study, we constructed pABs with metabolic glycoengineering and copper‐free click chemistry techniques. Based on the ability of MSCs‐ABs to regulate NAD metabolism, promote mitophagy, and treat fibrosis, pABs can further penetrate the hard matrix of fibrotic skin. Notably, pABs require only 50% of the injection force compared to MSC‐ABs while achieving deeper tissue distribution. This study is also the first known research on the issue of hard matrix in skin fibrosis. In summary, pABs have great potential for promoting metabolic therapy of fibrotic skin with hard matrix.

## 4 Conclusion

This study presents the development and therapeutic application of pABs, while elucidating the underlying mechanisms of AB‐mediated treatment for skin fibrosis. Through comprehensive in vitro investigations, we demonstrated that ABs modulate NAD metabolism and induce mitophagy in fibroblasts via the NAMPT/SIRT1/FOXO1 signaling axis. The excellent penetration effect of pABs on hard matrix and therapeutic effect on skin fibrosis were validated through human keloid xenograft models and bleomycin ‐induced skin fibrosis models. These findings establish a novel therapeutic strategy with significant potential for the treatment of skin fibrosis characterized by matrix rigidity.

## 5 Experimental Section

### Source of Clinical Samples

Under the ethical approval number 2021–785, matched keloid specimens and normal dermal tissues were prospectively collected from consenting patients (August 2023‐June 2024) for tissue staining, transplantation model construction, or extraction of primary cell KFs and NFs. This work recorded the basic information of the patients, the location of lesion, the scar area. Comprehensive clinicopathological data—including demographic characteristics, lesion topography, scar dimensions, and Modified Rodnan Skin Scores (mRSS; 0: unaffected skin; 1: 1–2 mm thickening/pinchable; 2: 3–5 mm induration/non‐pinchable; 3: >5 mm woody plaques)—were systematically cataloged (Table S1, Supporting Information). Medical ethics approval was obtained and informed consent forms were signed by all participants. Keloid and normal skin tissue were cut into rice grain‐sized tissue blocks, and the primary cell NFs and KFs were obtained for subsequent research.

### Primer Sequences and Experimental Materials

All oligonucleotide primers were custom‐designed using NCBI Reference Sequence databases and synthesized by Qingke Biotech Co., Ltd. (Hangzhou, China). The primer sequences are listed in Table S3, Supporting Information. All antibodies, chemical reagents, and detection kits are listed in Tables S4 and S5, Supporting Information.

### Cell Proliferation and Migration Ability

Cell proliferation and migration ability tests were measured according to previous research.^[^ ^44^, ^45^, ^46^ ^]^ The ECM‐Gel transwell method was used and crystal violet staining was performed to record the number of cells with different treatments, under a 20 × light microscope at 24, 48, and 96 h.

### Cell Uptake

PKH26‐labeled ABs were co‐cultured with KFs at a concentration of 1 µg mL^−1^ for 1, 2, 3, and 24 h. Then the culture medium was removed and washed three times with PBS. Subsequent steps involved fixing the cells with 4% paraformaldehyde for 20 min, followed by staining of both the cytoskeleton and cell nuclei. The process of KFs uptaking ABs was characterized under confocal microscopy (Leica STELLARIS 5).

### Cellular Mitochondrial Function Test

To detect mitochondrial membrane potential, cells (1 × 10⁶) were seeded in a 6‐well plate and incubated with 1 mL of pre‐warmed JC‐1 staining solution at 37 °C for 20 min. Fluorescence images were acquired using an inverted fluorescence microscope (Olympus IX73). For intracellular reactive oxygen species (ROS) detection, cells (1 × 10⁶) were treated with 1 mL of DCFH‐DA (10 µM) and incubated at 37 °C for 20 min. Fluorescence imaging was performed under the same conditions.

### Mitophagy Measurement

Mitophagy was assessed using a dual‐staining approach. Cells were labeled with MitoTracker Green (mitochondria), LysoTracker Red (lysosomes), and Hoechst 33 342 (nuclei), followed by observation under a laser confocal microscope. Additionally, Western blotting was performed to quantify key mitophagy‐related proteins, including PINK1, PARKIN, LC3, and p62, to further validate autophagic flux.

### NAD^+^ Level Measurement

For cellular NAD^+^ measurement, 1 × 10⁶ cells were lysed in 200 µL of ice‐cold extraction buffer, homogenized, and centrifuged at 12,000 rpm (4 °C, 10 min). The supernatant was collected for analysis. In a 96‐well plate, 20 µL of the supernatant was mixed with ethanol dehydrogenase working solution and incubated at 37 °C in the dark for 30 min. Absorbance was measured at 450 nm. For the tissue samples, after washing the tissue with pre‐cooled PBS, weigh 20 mg of the tissue sample, cut it into pieces, add 400 µL of extraction solution homogenate, then centrifuge at 12 000 rpm and 4 °C for 10 min, the supernatant was subjected to the same NAD^+^ detection protocol.

### Fabrication and Characterization of ABs/pABs

MSCs‐ABs were prepared according to the previous research^[^ ^47^ ^]^ and partially modified. Briefly, MSCs were treated with 5 µM staurosporine in high‐glucose medium for 6–12 h to induce apoptosis. The resulting apoptotic cells were centrifuged at 300 rpm for 10 min, and the supernatant was collected. A secondary centrifugation step (3000 rpm, 10 min, 4 °C) was performed to pellet the ABs. To remove residual apoptotic cells and large debris, the suspension was filtered through a 1‐µm pore‐size membrane. The purified ABs were resuspended in PBS and stored at −20 °C for long‐term use. To obtain pABs, first, 10 µg of MSCs‐ABs with/without 100 µM Ac_4_ManNAz in 1 mL PBS at 37 °C, 48 h, 3000 rpm, 10 min. Take the precipitate and suspend it in PBS to obtain AcMz‐ABs. Solution of the type I collagen (100 mg) in DMF (1.5 mL) was added HATU (250 mg) and DIEA (150 mg), followed by adding DBCO‐NH_2_ (100 mg, 0.8 mmol). Then mixture was stirred at room temperature for 24 h, after which time the reaction mixture was diluted with H_2_O and extracted with ethyl acetate. The org layer was dried (Na_2_SO_4_) and concentrated in vacuo. Then the residue was separated and purified by High Performance Liquid Chromatography (HPLC) to obtain DBCO‐type I collagenase. Finally, 100 µg AcMz‐ABs and 1 mg DBCO‐type I collagenase were treated with a stirrer in 1 mL PBS at 37 °C, 50 rpm, 24 h, then 3000 rpm, 10 min. The precipitate was taken and suspended in PBS to obtain pABs, which were stored at −20 °C for future use. The morphology of ABs and pABs was observed through TEM, and the characteristic peaks of ABs, pABs, and other groups were detected through FTIR. The hydrodynamic diameter distribution and zeta potential of pABs were determined via DLS. Western blotting was performed to assess CD105 (a stem cell surface marker), Caspase 3 and Cleaved Caspase 3 (apoptosis markers).

### Tissue Permeability Measurement

6‐week‐old male nude mice were selected and human keloid tissue was subcutaneously implanted in the bilateral scapular area. After 28 days, the nude mice were euthanized and the keloid tissue was evaluated using HE and Masson staining. Tissue collagen deposition levels were detected through Masson and Sirius Red staining. To test the permeability of pABs to keloid, 1 × 10^6^ DiR‐labeled ABs and pABs were first injected into the back of nude mice transplanted with keloid. 1 week after injection, COL1A1/DAPI immunofluorescence (IF) staining was performed to observe the quantity and area of pABs penetrating the matrix. The change of thrust required for the syringe to penetrate the substrate was detected with a push‐pull force meter. The fluorescence signal intensity of DiR – labeled pABs after subcutaneous injection on day 0, 3, and 7 was detected using animal live imaging system.

### Skin Fibrosis Mouse Model

In this study, all animal‐related experiments were conducted under the guidance of the Experimental Animal Ethics Committee of the First Affiliated Hospital of Zhejiang University School of Medicine (2024‐1204). BLM is widely used as an inducer for the skin fibrosis model. As previous research,^[^ ^48^ ^]^ 6‐week‐old male BALB/c mice were selected, and BLM was injected subcutaneously at a dose of 1 mg kg^−1^ for 3 weeks. On the 21st day, the mice were euthanized and the diseased tissue was taken. Starting from the 22nd day, ABs/pABs (5 mg kg^−1^) were injected subcutaneously daily. On day 28, the mice were euthanized and the diseased tissue was taken for HE, Masson, Siron Red staining and ROS staining. The expression of α‐SMA, COL1A1, and fibronectin were detected, and Image J software was used to randomly calculate the area of 15 IF‐positive areas for statistical analysis. Body weight, dietary intake, and success and survival rates of model mice are listed in Table S2, Supporting Information.

### Transcriptome Analysis

Total RNA was extracted from KFs treated with ABs, and its integrity and purity were verified by agarose gel electrophoresis. Magnetic beads were used to selectively enrich mRNA and disperse it into short fragments. Reverse transcriptase was used to transcribe mRNA into cDNA, and DNA polymerase was used to synthesize double‐stranded DNA using cDNA templates. DsDNA fragments were inserted into the sequencing vector to construct a library. Illumina was used to sequence the library and perform GO/KEGG enrichment analysis on DEGs.

### Statistical Analysis

Statistical analysis was conducted using SPSS 22.0 software. The data was shown as mean ± standard deviation, and Student's *t*‐test and analysis of variance were used for inter‐group comparison to determine the significance of differences. All data comes from at least 3 independent experimental replicates. It is considered statistically significant when the *p*‐value is less than or equal to 0.05.

## Conflict of Interest

The authors declare no conflict of interest.

## Author Contributions

S.Q., S.D., and C.G. contributed equally to this work. S.Q., S.D., and C.G. conceived and designed the research. S.Q. and C.G. wrote the manuscript. J.X., S.D., and X.S. provided funding support. W.C. provided supervision and project administration. S.Q. and C.G. performed most of the experiments in this study and prepared the figures. J.H., W.W., J.P.,Y.S., and M.X. were crucial for clinical specimens, fibroblast isolation, western blotting and mouse model creation.

## Supporting information



Supporting Information

## Data Availability

The data that support the findings of this study are available in the supplementary material of this article.
