# Supplementary material for: Apoptotic Bodies Restore NAD and Mitochondrial Homeostasis in Fibroblasts
Source: Adv Sci (Weinh). 2025 May 19;12(29):e15691. doi: 10.1002/advs.202415691 (PMC12362740; doi:10.1002/advs.202415691)
Supplement: Supplementary file 1 — Supporting Information [file ADVS-12-e15691-s001.docx]

Supporting Information for

**Apoptotic Bodies Restore NAD and Mitochondrial Homeostasis in Fibroblasts**

Shutong Qian^1^†, Siya Dai^1^†, Chunyi Guo^1^†, Wenjun Wang^1^, Jiajia Pang^1^, Yichen Shen^1^, Mingyuan Xu^1^, Jie Hu^1^*, Wenguo Cui^3^*, Xiaoming Sun^2^*, Jinghong Xu^1^*

**Affiliations**

Shutong Qian^1^, Siya Dai^1^, Chunyi Guo^1^, Wenjun Wang^1^, Jiajia Pang^1^, Yichen Shen^1^, Mingyuan Xu^1^, Jie Hu^1^*, Jinghong Xu^1^*

^1^Department of Plastic Surgery, The First Affiliated Hospital, Zhejiang University School of Medicine, Hangzhou 310003, P. R. China.

E-mail: Jiehu0101@zju.edu.cn; doctorxjh@zju.edu.cn

Xiaoming Sun^2*^

Department of Plastic and Reconstructive Surgery, Shanghai Ninth People’s Hospital, Shanghai JiaoTong University School of Medicine, Shanghai 200011, P. R. China.

E-mail: drsunxm@126.com

Wenguo Cui^3*^

Department of Orthopaedics, Shanghai Key Laboratory for Prevention and Treatment of Bone and Joint Diseases, Shanghai Institute of Traumatology and Orthopaedics, Ruijin Hospital, Shanghai Jiao Tong University School of Medicine, 197 Ruijin 2nd Road, Shanghai 200025, P. R. China.

E-mail: wgcui@sjtu.edu.cn

†These authors contributed equally to this work.


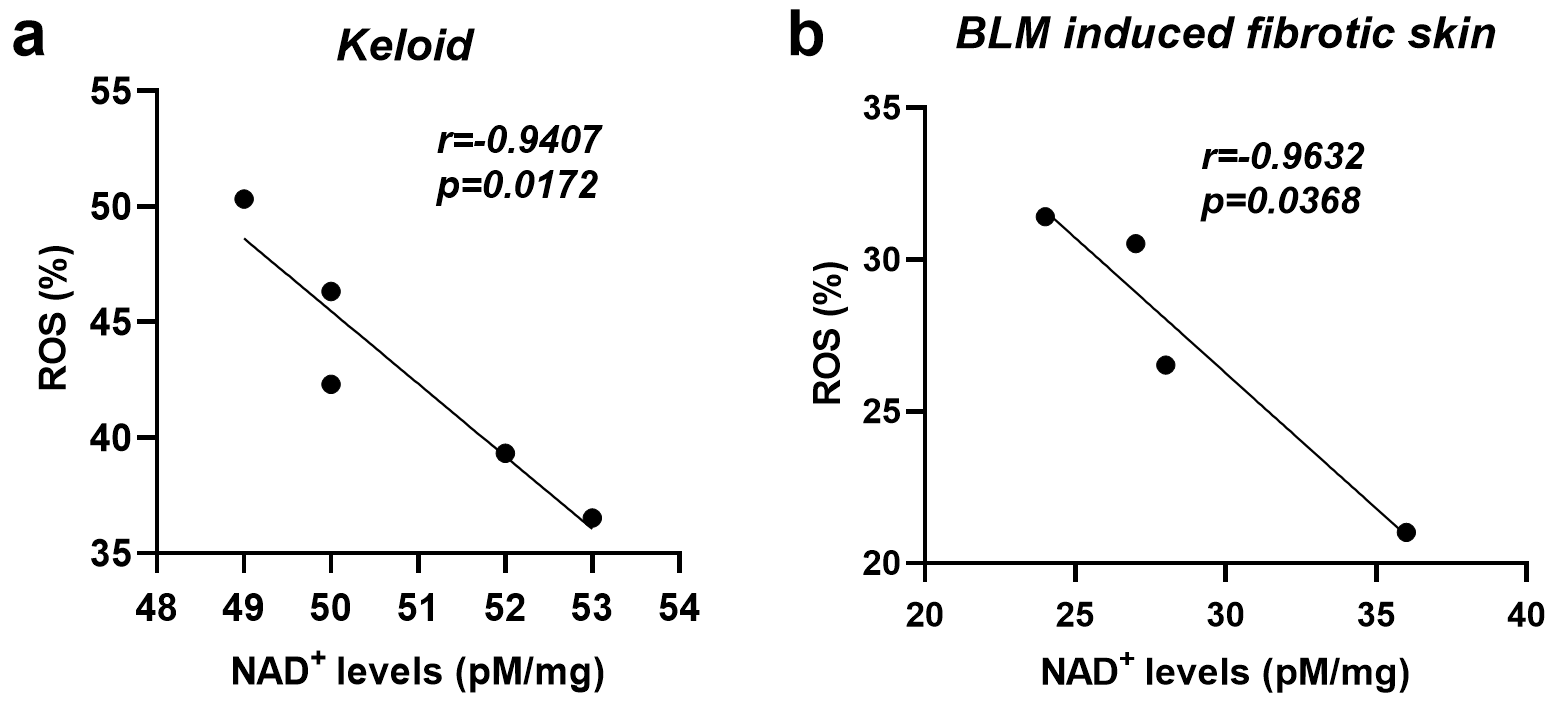


**Figure S1. Characterization of ABs.** a) TEM images of ABs. Scale bars, 100 μm. b) Zeta potential analysis of cell membrane surface of ADSCs and ABs (n = 5). c) DLS


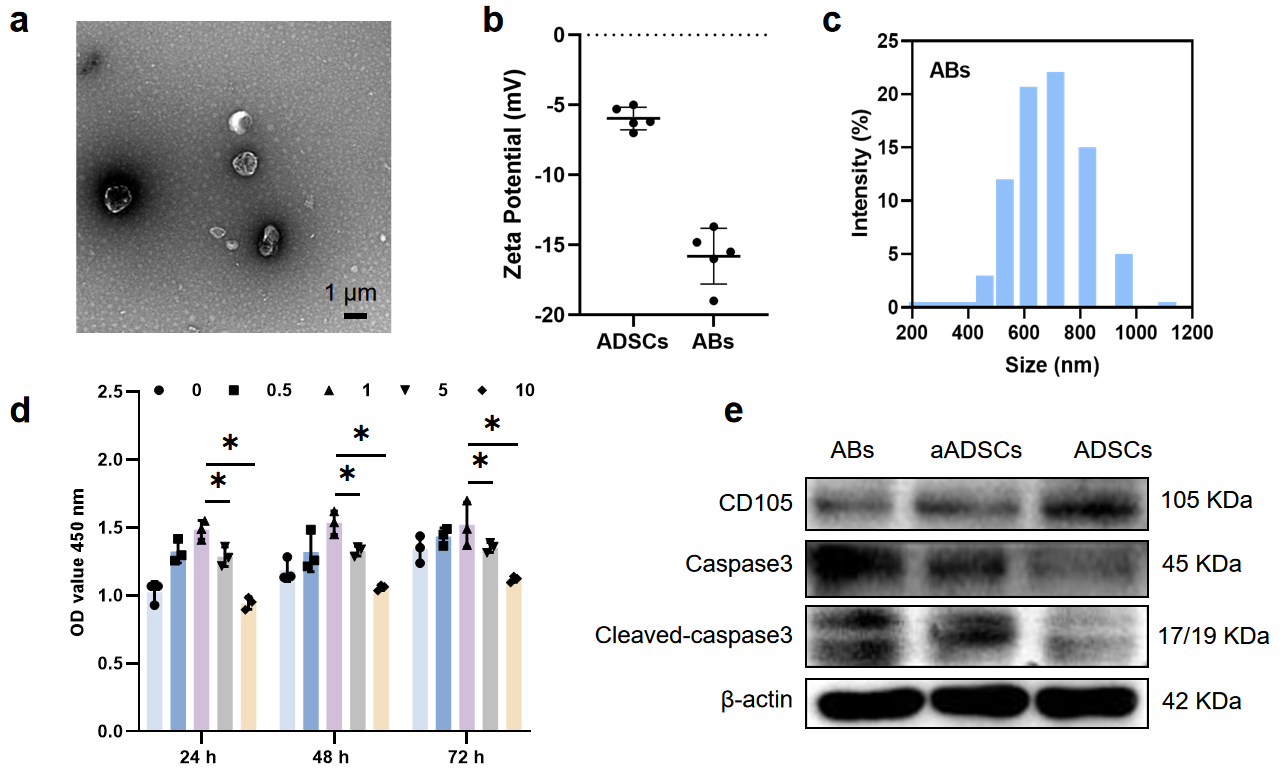


**Figure S2. Characterization of ABs.** a) TEM images of ABs. Scale bars, 100 μm. b) Zeta potential analysis of cell membrane surface of ADSCs and ABs (n = 5). c) DLS analysis of ABs. d) OD values of KFs after co-culturing with different concentrations and time points of ABs (n = 3). e) Protein expression of apoptosis markers Caspase3 and Cleaved Caspase3, and stem cell surface marker CD105, in ABs, apoptotic ADSCs (aADSCs), and ADSCs. * *p*<0.05.


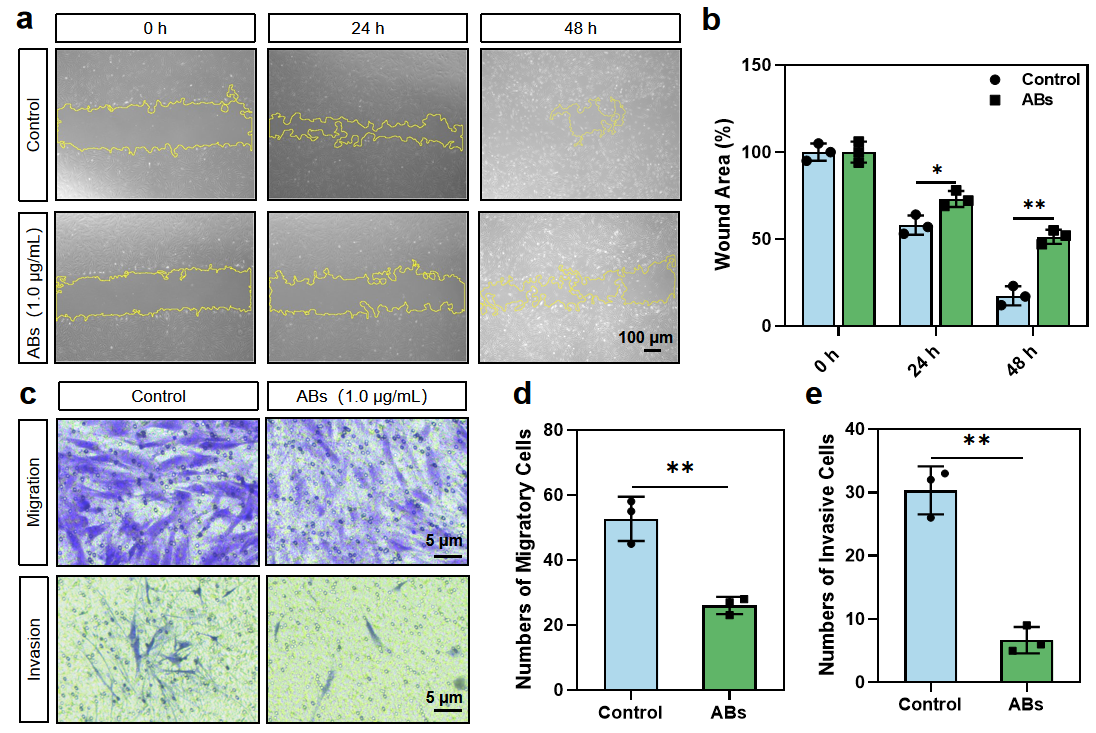


**Figure S3. ABs inhibit the migration and invasion ability of KFs.** a) Typical cell scratch test images of different groups at different time points. Scale bars, 100 μm. b) Statistical analysis of wound area in the two groups (n = 3). Scale bars, 5 μm. c) Typical cell migration and invasion images of different groups at 24 h. d-e) Statistical analysis of numbers of migratory and invasive cells of the two groups (n = 3). **p*<0.05, ***p*<0.01.


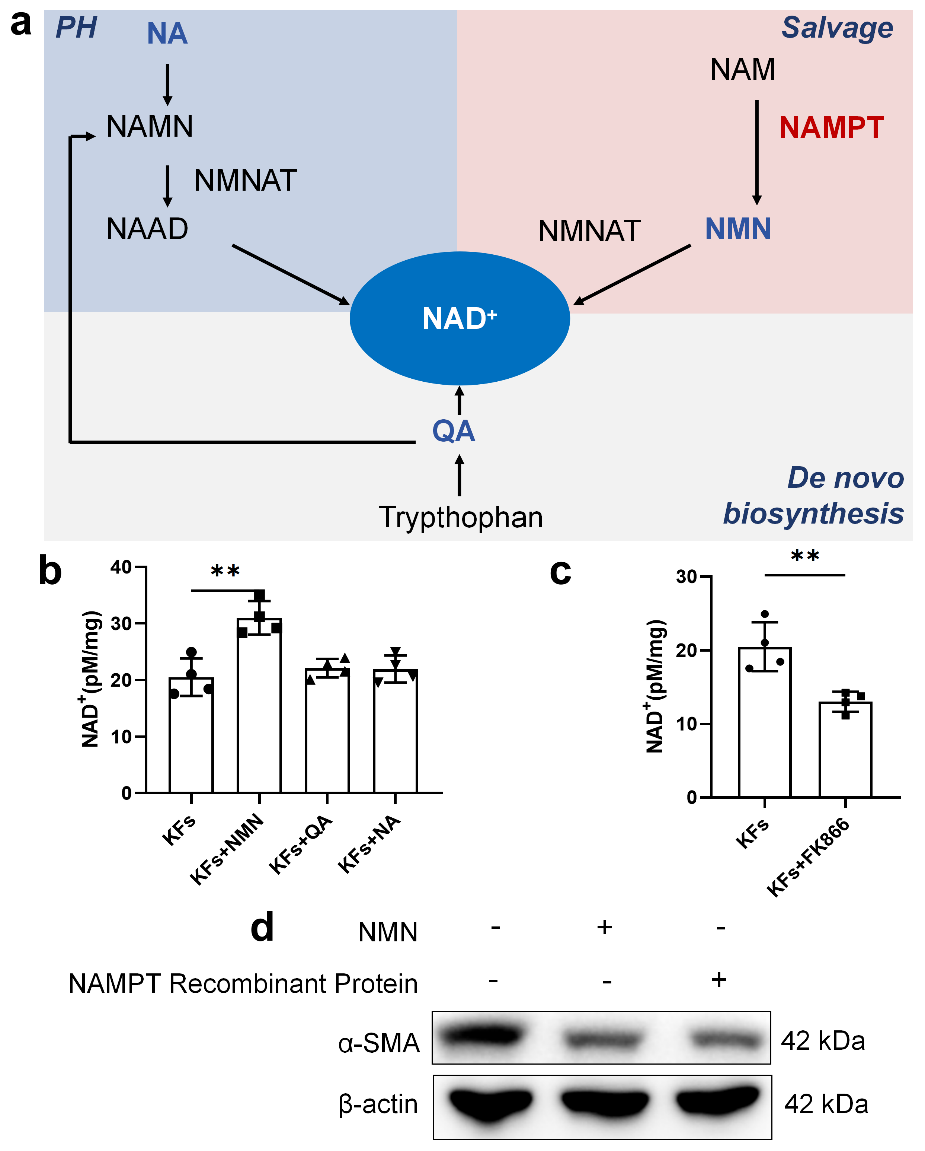


**Figure S4. The NAD metabolism in KFs mainly relies on the salvage synthesis pathway.** a) Schematic diagram of three pathways for synthesis of NAD^+^. b) NAD^+^ level of KFs treated with different NAD^+^ precursor substances (n = 4). c) NAD^+^ level of KFs treated with or without FK866 (n = 4). d) Western Blot image of KFs treated with NMN or NAMPT protein. ***p*<0.01.


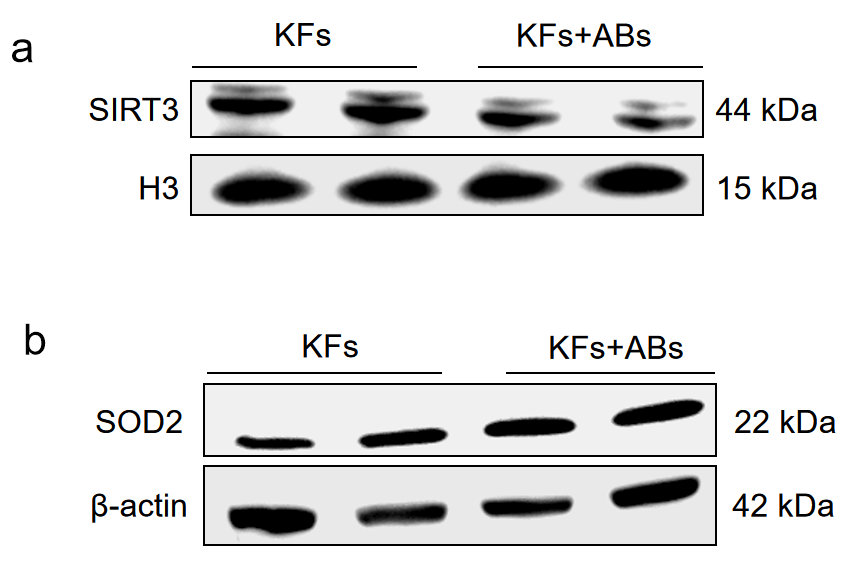


**Figure S5. Protein expression of SIRT3 and SOD2 in different groups.** a-b) Protein expression of SIRT3 and SOD2 in different groups.


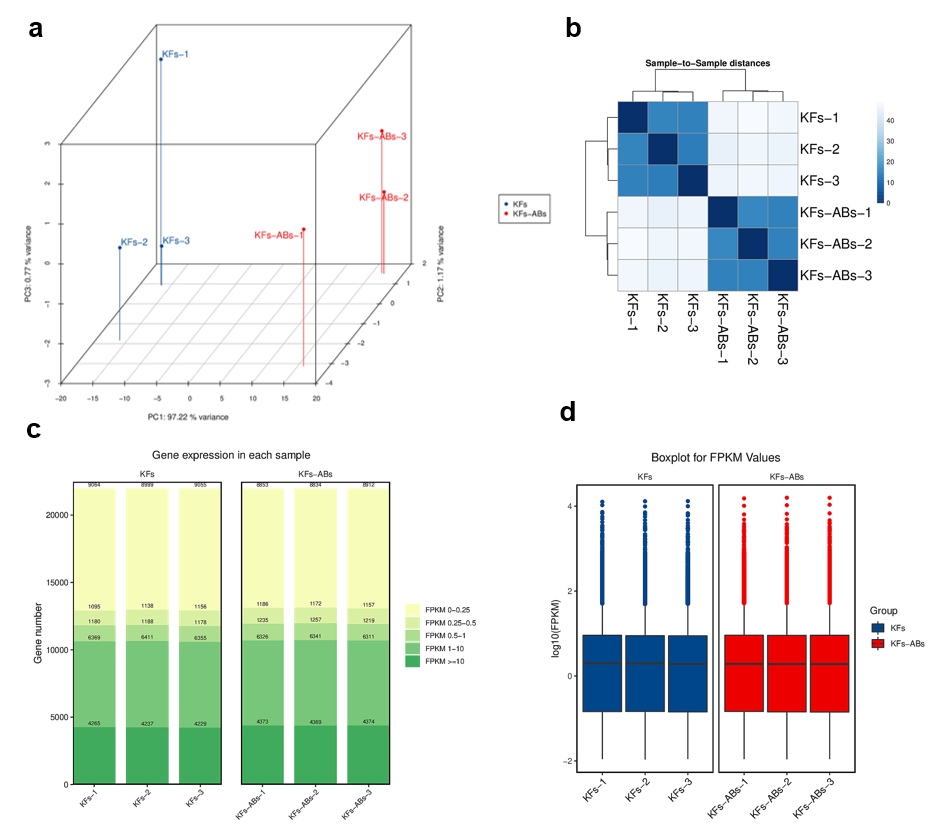


**Figure S6. Gene expression in each sample.** a-b) Principal component analysis (PCA) and sample-to-sample distances heatmap in different groups. c) Gene expression at different fragments per kilobase of exon model per million mapped fragments (FPKM) level in each sample. d) Boxplot for FPKM values.


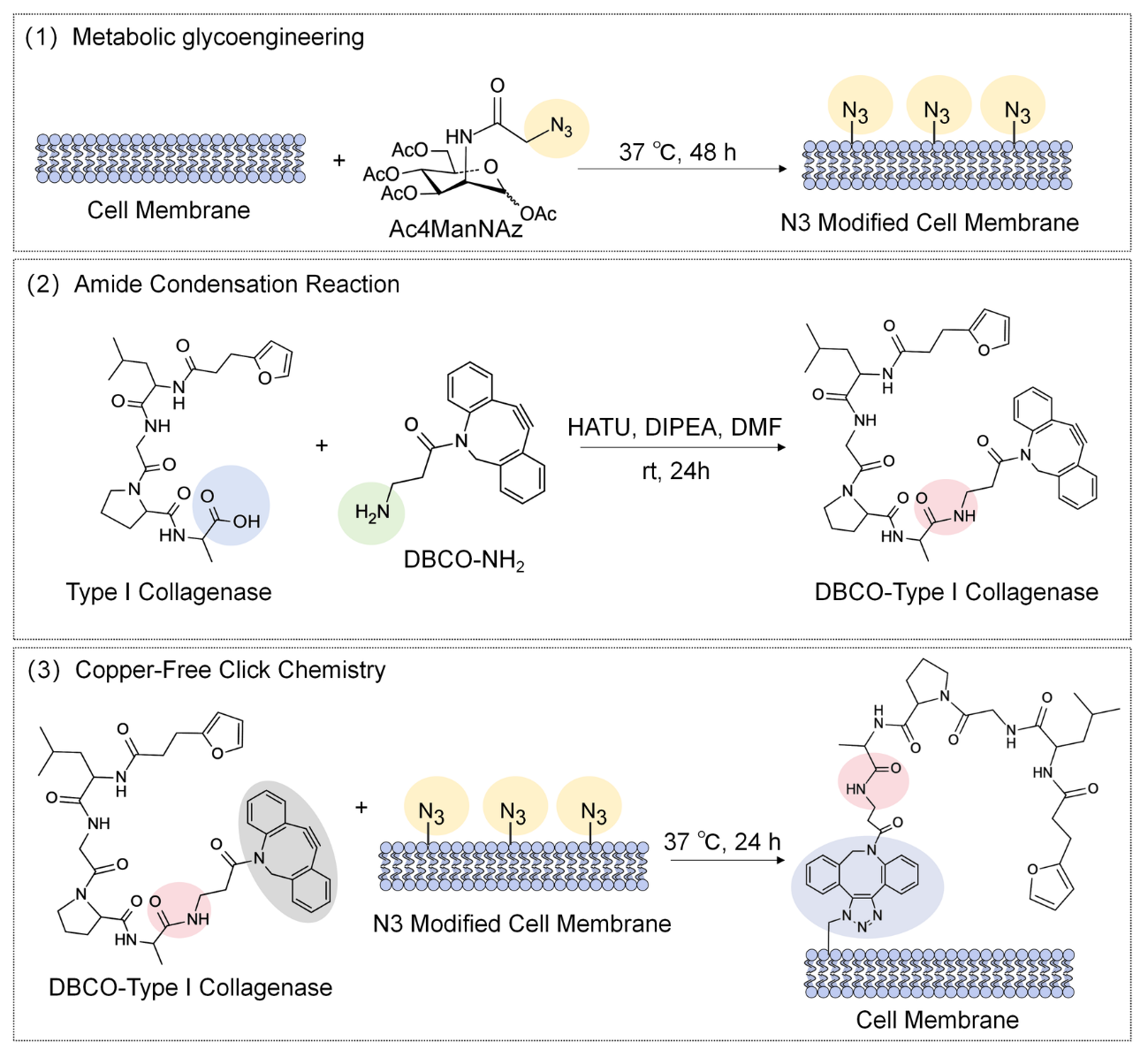


**Figure S7. Scheme of fabrication process of pABs.**


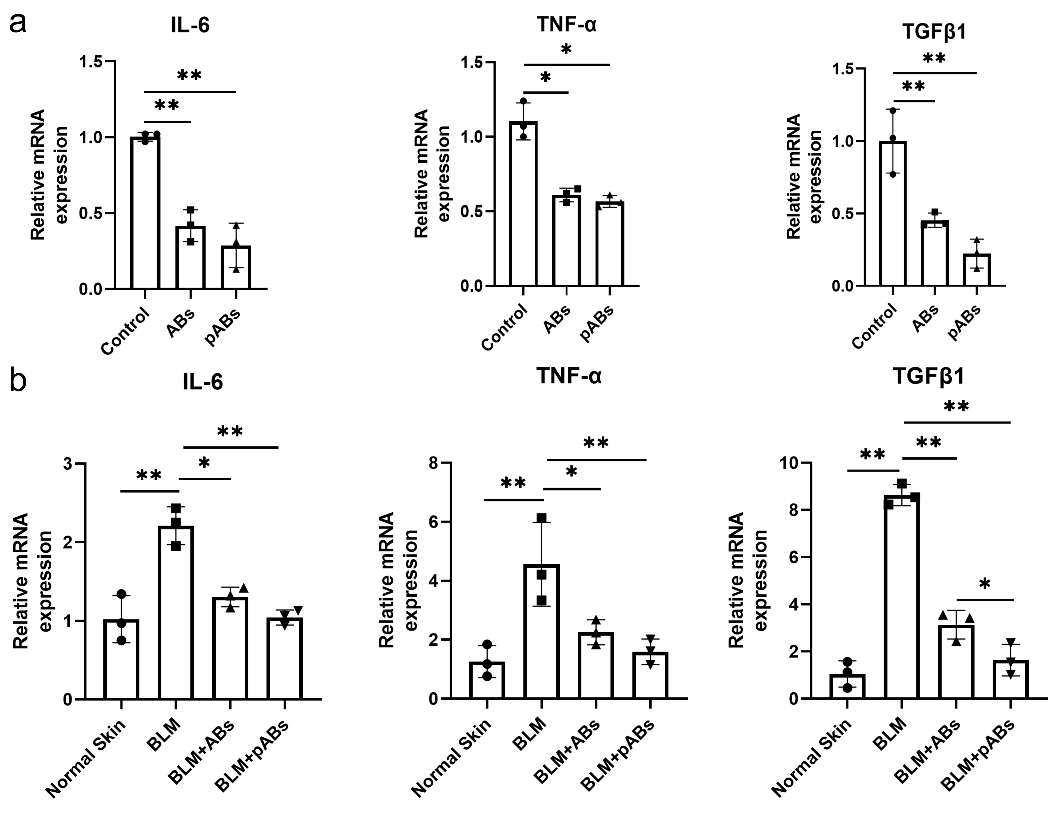


**Figure S8. mRNA expression of TNF-α, IL-6 and TGFβ1.** a-b) mRNA expression of TNF-α, IL-6 and TGFβ1 in keloid transplantation model and bleomycin-induced skin fibrosis model. **p*<0.05，***p*<0.01.

**Table S1: Source of human keloid.**

| **Patient ID** | **Age (years)** | **Gender** | **Site of biopsy** | **Rodnan Score** | **Scar Area**  **(cm^2^)** | **Use in This Study** |
| --- | --- | --- | --- | --- | --- | --- |
| #1 | 25 | Female | Ear | 2 | 3.1 | Extraction of primary cells; Tissue Staining |
| #2 | 58 | Female | Ear | 3 | 8.0 | Extraction of primary cells; Tissue Staining |
| #3 | 26 | Female | Ear | 3 | 12.6 | Extraction of primary cells; Tissue Staining |
| #4 | 36 | Female | Forearm | 3 | 8.4 | Extraction of primary cells; Tissue Staining |
| #5 | 41 | Male | Ear | 3 | 6.2 | Extraction of primary cells; Tissue Staining |
| #6 | 37 | Female | Ear | 3 | 10.2 | Transplantation |
| #7 | 32 | Male | Ear | 3 | 8.9 | Transplantation |
| #8 | 29 | Female | Ear | 3 | 7.6 | Transplantation |

**Table S2: Body weight, dietary intake, and success and survival rates of model mice.**

| **BLM-induced fibrosis model** | | | | | | |
| --- | --- | --- | --- | --- | --- | --- |
| Number | #1 | #2 | #3 | #4 | #5 | #6 |
| Weight | 28.9 | 29.4 | 30.1 | 28.3 | 30.3 | 29.4 |
| Feed consumption/1 day (g) | 15.4 | 13.6 | 16.1 | 12.2 | 17.6 | 13.2 |
| Water intake/1 day (mL) | 30 | 27 | 27 | 28 | 32 | 21 |
| Success | √ | √ | √ | √ | √ | × |
| Survival | √ | √ | √ | √ | √ | √ |
| **Keloid transplantation nude mouse model** | | | | | | |
| Number | #1 | #2 | #3 | #4 | #5 | #6 |
| Weight | 28.2 | 28.5 | 30.6 | 29.1 | 31.0 | 29.4 |
| Weight after transplantation | 29.2 | 29.5 | 31.6 | 30.1 | 32.0 | 30.4 |
| Feed consumption/1 day (g) | 14.7 | 15.3 | 12.2 | 13.5 | 14.6 | 18.2 |
| Water intake/1 day (mL) | 29 | 29 | 26 | 28 | 28 | 27 |
| Success | √ | √ | √ | √ | × | × |
| Survival | √ | √ | √ | √ | √ | √ |

**Table S3: Primer used for real-time quantitative PCR (RT-qPCR)**

| Genes | | Source | Forward primer | Reverse primer |
| --- | --- | --- | --- | --- |
| *NAMPT* | | Human | AGGGTTACAAGTTGCTGCCACC | CTCCACCAGAACCGAAGGCAAT |
| *JUN* | | Human | CCTTGAAAGCTCAGAACTCGGAG | TGCTGCGTTAGCATGAGTTGGC |
| *SOD2* | Human | | CTGGACAAACCTCAGCCCTAAC | AACCTGAGCCTTGGACACCAAC |
| *CCN1* | Human | | GGAAAAGGCAGCTCACTGAAGC | GGAGATACCAGTTCCACAGGTC |
| *CCN2* | Human | | CTTGCGAAGCTGACCTGGAAGA | CCGTCGGTACATACTCCACAGA |
| *FGF1* | Human | | ATGGCACAGTGGATGGGACAAG | TAAAAGCCCGTCGGTGTCCATG |
| *PDGFD* | Human | | GCGGCTTCACTCTCAGGAGAAT | CTTGTGTCCACACCATCGTCCT |
| *IL-6* | Human | | AGACAGCCACTCACCTCTTCAG | TTCTGCCAGTGCCTCTTTGCTG |
| *TNF-α* | Human | | CTCTTCTGCCTGCTGCACTTTG | ATGGGCTACAGGCTTGTCACTC |
| *TGF**β1* | Human | | TACCTGAACCCGTGTTGCTCTC | GTTGCTGAGGTATCGCCAGGAA |

**Table S4: Source of materials**

| **Materials** |  |
| --- | --- |
| NAD^+^/NADH Assay Kit with WST-8 | Beyotime，S0175 |
| Mitochondrial membrane potential assay kit with JC-1 | Beyotime，C2006 |
| Type I collagenase | Gibco，17100017 |
| CCK8 Kit | Solarbio，CA1210 |
| Ac4ManNAz | Gibco，GC60038 |
| Cyanine 5 DBCO | Aladdin，C171368 |
| FK866 | Gibco，GC14308 |
| Fast Blocking Western | GeneFist，GF1815 |
| Stripping Buffer | Applygen，P1650 |
| PVDF Membrane | Millipore，IPVH00010 |
| Total RNA Isolation Kit | ESScience，RN001 |
| Protein Ladder | Thermo，26616 |
| Rotenone | Glpbio，GC16775 |
| SuperEnhanced chemiluminescence detection reagents | Applygen，P1060 |
| Reactive Oxygen Species Assay Kit | Beyotime，S0033S |
| Push-pull Force Gauge | AIGU，NK-10200300500 |
| Lyso-Tracker-Red | Beyotime，C1046 |
| Mito-Tracker Green | Beyotime，C1048 |
| Hoechst 33342 | Beyotime，C1027 |
| Actin-Tracker Red-594 | Beyotime，C2205S |
| EX-527 | Beyotime，SC0281 |

**Table S5: Source of WB antibodies.**

| **Antibodies** | | |
| --- | --- | --- |
| NAMPT | Rabbit | Affinity, DF6059 |
| SOD2 | Rabbit | Immunoway, YT5575 |
| α-SMA | Rabbit | Cell Signaling Technology, 19245T |
| COL1A1 | Rabbit | Cell Signaling Technology, 72026T |
| FOXO1 | Rabbit | Immunoway, YP0113 |
| Acetyl-FOXO1 | Rabbit | Immunoway, YK0110 |
| SIRT1 | Rabbit | Immunoway, YT4302 |
| SIRT3 | Rabbit | Immunoway, YT4304 |
| p62 | Rabbit | Abcam, ab109012 |
| LC3B | Rabbit | Abcam, ab192890 |
| β-actin | Rabbit | Immunoway, YT0099 |
| Histone H3 | Rabbit | Immunoway, YT2163 |
| PINK1 | Rabbit | Abcam, ab216144 |
| PARKIN | Mouse | Abcam, ab77924 |
| Caspase3 | Rabbit | Abcam, ab184787 |
| Cleaved-caspase3 | Rabbit | Abcam, ab2302 |
| CD105 | Rabbit | Abcam, ab231774 |
